# Accuracy of Pretreatment Ultrasonography Assessment of Intra-Abdominal Spread in Epithelial Ovarian Cancer: A Prospective Study

**DOI:** 10.3390/diagnostics11091600

**Published:** 2021-09-02

**Authors:** Agnieszka Tomasińska, Maciej Stukan, Michał Badocha, Aleksandra Myszewska

**Affiliations:** 1 Department of Gynecologic Oncology, Gdynia Oncology Center, Pomeranian Hospitals, 81-519 Gdynia, Poland; amyszewska@szpitalepomorskie.eu; 2 Department of Physical Chemistry, Faculty of Chemistry, Gdańsk University of Technology, 80-233 Gdańsk, Poland; micbadoc@pg.edu.pl

**Keywords:** ovarian cancer, ultrasonography, preoperative, staging, accuracy, prediction, residual disease, surgery, complexity, mesentery root

## Abstract

The aim of this study was to test the accuracy of ultrasonography performed by gynecological oncologists for the preoperative assessment of epithelial ovarian cancer (EOC) spread in the pelvis and abdominal cavity. A prospective, observational cohort study was performed at a single tertiary cancer care unit. Patients with suspected EOC were recruited and underwent comprehensive transvaginal and abdominal ultrasonography performed by a gynecological oncologist. Sixteen intra-abdominal localizations and parameters were assessed using ultrasonography and compared with surgical-pathological status (reference standard). Sensitivity, specificity, positive and negative predictive values, and overall accuracy were calculated. Differences were analyzed using Fisher’s exact and chi-square tests. Ultimately, we included 132 patients (median age 62 years), of whom 67% were in stage IIIC–IVB and 72% had serous cancer. Overall prediction accuracies for the involvement of the omentum, small bowel mesentery root, and frozen pelvis, and detecting ascites were >90%. Detecting the involvement of the pelvis peritoneum, liver and spleen hilum, and rectosigmoid colon, and predictions of disease stage and residual disease had overall accuracies of 80–90%. The lowest accuracy was for involvement of the abdominal peritoneum (69%) and diaphragm peritoneum (right 71%; left 75%) and surgical complexity prediction (77%). Stratification of results by presence or absence of ascites revealed significantly higher specificity of ultrasonography (clinically meaningful) for assessments of the abdominal/pelvic peritoneum, spleen hilum, and rectum wall, if there were ascites. A gynecological oncologist, experienced in surgery and sonology, performing comprehensive ultrasonography on patients with EOC can accurately detect intraperitoneal lesions and recognize critical disease manifestations and predict stage, surgical complexity, and residual disease, which allow accurate qualification of patients for primary debulking surgery or neoadjuvant chemotherapy.

## 1. Introduction

Epithelial ovarian cancer (EOC) is the leading cause of death among all gynecological cancers in developed countries, with most patients presenting with advanced-stage tumors, which are defined by the spread of the disease outside the pelvis (International Federation of Obstetrics and Gynecology (FIGO) stages III and IV—more than two-thirds of patients at diagnosis) [1]. The estimated number of new ovarian cancer cases in Europe in 2020 was 39,239 with 27,135 deaths [2]. The standard treatment includes debulking surgery followed by adjuvant chemotherapy, which is the preferred strategy [1]. However, primary cytoreduction is reasonable in patients with predicted complete cytoreduction (removing all macroscopic disease) and estimated low risk of surgical complications. For advanced EOC, where resection to residual disease of 1 cm or less is unlikely or the risk of complication is high, neoadjuvant chemotherapy and interval debulking surgery are associated with improved survival and reduced perioperative morbidity compared with those of primary debulking surgery (PDS) [3]. Clinical procedures should be specifically tailored to each patient with EOC and depend on possibilities of complete cytoreduction, surgical skills and experience of the physician, institution infrastructure, and patient characteristics [4]. To estimate the extent of the disease and resectability, different imaging modalities are used. To predict the risk of major complications concerning extensive cytoreduction, numerous patient-related factors need to be considered, which include the use of frailty indexes [5,6,7].

The preferred technique for detection of ovarian malignancies is transvaginal ultrasound because of its availability, high resolution, and lack of ionizing radiation [8]. Expert sonologists can predict ovarian malignancy with high probability [9]. Currently, the first-line imaging modality for staging, selecting treatment options, and assessing disease response in EOC is computed tomography (CT) of the abdomen and pelvis [1]. However, for the prediction of disease stage, sensitivity and specificity of CT have been reported to be 50% and 92%, respectively, and most mesenteric and small-bowel implants are not detected [10]. During the presurgical workup, whole-body diffusion-weighted magnetic resonance imaging can be useful, where it has shown to be more accurate for assigning the correct stage to patients with EOC compared with CT [11]. 

Ultrasonography performed by an expert examiner may be a new strategy for evaluating the intra-abdominal spread of disease in EOC [12,13,14,15]. Because of the low cost and high availability of examination and the comparable—or even better—accuracy, transvaginal and transabdominal ultrasonography may be considered an alternative modality to CT scanning [16,17].

The aim of the present study was to test the accuracy of ultrasonography performed by gynecological oncologists for the preoperative assessment of EOC spread in the pelvis and abdominal cavity.

## 2. Materials and Methods

### 2.1. Study Design

This was a prospective, observational cohort study performed at a single tertiary cancer care unit at the Gynecologic Oncology Department in Gdynia Oncology Center, Szpitale Pomorskie, Gdynia, Poland, from June 2018 to February 2021. The department is certified by the European Society of Gynecological Oncology (ESGO) for the treatment of patients with advanced EOC.

Ultrasonography data were acquired prospectively before surgery, saved, and remained unchanged. Data on surgery and histology were collected and saved after the procedure.

Study data were collected and managed using REDCap electronic data capture tools [18,19], hosted at Szpitale Pomorskie, Gdynia Oncology Center, Poland. No patient-identifying data were collected.

A time interval between ultrasonography and surgery of up to 30 days was permitted for patient inclusion in the study.

### 2.2. Participants

Eligible subjects were consecutive patients with suspected EOC who were referred to the Gynecology Oncology Department for additional diagnosis and treatment. Inclusion criteria were age > 18 years, presence of a suspicious adnexal mass that was detected in a transvaginal ultrasound performed at our center, feasible for surgery (open or laparoscopic evaluation), agreed to undergo comprehensive transvaginal and abdominal ultrasonography. Prediction of malignancy was based on the examiner’s subjective assessment, or if ambiguous in appearance, multivariable predictive models described elsewhere were used [20,21]. In addition, patients without an apparent adnexal mass who were otherwise suspected for primary peritoneal cancer were included. Exclusion criteria were no surgery (due to poor patient status, severe comorbidities, or extra-abdominal disease), diagnosis of EOC based on a percutaneous biopsy, diagnostic laparoscopy but without the possibility to fully explore the pelvis and abdominal cavity, no cancer or non-ovarian malignancy in the final histopathological examination. 

### 2.3. Imaging Technique (Index Test)

Ultrasound examination using endovaginal and convex probes was performed at our department as a standard procedure in every patient with suspected EOC. Ultrasonography was performed by A.T., who was a resident in gynecological oncology, and M.S. and A.M. who were gynecological oncologists working as specialists for 6 and 4 years, respectively. In Poland, gynecologists are obligatorily trained in pelvic ultrasonography during the residency period. Thus, every gynecologist is able and allowed to perform pelvic ultrasonography. Furthermore, during the gynecological oncology training, M.S. and A.M. participated in additional sonological courses. M.S. underwent specific training in gynecological oncology ultrasonography. A.T. underwent additional training in pelvic floor ultrasonography, which included assessment of urinary bladder and rectum. However, surgical treatment is their primary specialty, which is why examiners are rated as level 2 sonologists according to the European Federation of Societies for Ultrasound in Medicine and Biology classification [22]. Additionally, each expert examiner was involved in the surgical treatment of the diagnosed patients. A Philips HD15^®^ ultrasound machine (Philips Healthcare, Best, The Netherlands) equipped with 2.4–5 MHz convex and 5–9 MHz endovaginal probes was used. This device is standard equipment in our department. Patients did not undergo any preparation (e.g., enema or fasting), and no contrasts were used. The diagnostic test protocol was based on the methodology described elsewhere [23,24]. 

The anatomical areas assessed for involvement and ultrasound criteria are described and presented, with examples, in Table 1. Initially, we assessed the retroperitoneal space around the aorta, inferior vena cava, and pelvic iliac vessels to search for enlarged lymph nodes. However, we did not include this parameter for analysis because surgical and histological assessments of this parameter varied considerably among the included patients. 

We performed examinations in grayscale to evaluate the above locations and lesions. Doppler was turned on only to verify the location of blood vessels. When performing the ultrasound, we usually perform the examination actively—making pressing movements with the ultrasound probe or the other hand—dynamic ultrasonography as described elsewhere [24]. We applied this method for all ultrasound examinations, including abdominal cavity scanning (not only the pelvis). This allowed the visualization of an infiltrated omentum, the structure, and mobility of the mesentery of the small bowel, as well as the rectum and peritoneum of the pouch of Douglas. Examinations began with an assessment of the pelvis using an endovaginal probe, then the lower, mid, and upper abdomen was assessed using the convex probe. Scanning the abdomen first and the pelvis second was permitted. The examiner detected the presence or absence of disease in the tested localizations. 

Predictions of disease stage, surgical complexity, and residual disease were calculated based on ultrasonography for each patient before surgery. The FIGO system was used for staging EOC [27]. To simplify the categorization and calculations, we divided EOC stages into two entities: early (FIGO I–IIA) and advanced (FIGO IIB–IVB). Surgical complexity was calculated as described elsewhere [26]. For prediction of residual disease, stratification into early or advanced stages based on ultrasonography was not performed (i.e., residual disease status prediction after the surgery was calculated independently, regardless of whether it was early or advanced disease because it cannot be estimated based on imaging only).

Abdominal and/or chest CT was performed only in selected cases, e.g., those with ambiguous ultrasound findings.

### 2.4. Surgery and Histology (Reference Standard)

Exploration of all predefined sites in the pelvis and abdominal cavity was performed during surgery via the laparotomy approach, with an incision made along the longitudinal line from the symphysis pubis to the xiphoid process. All macroscopic findings were recorded on the database. Additionally, confirmation was obtained from the histology report. In patients who only underwent diagnostic laparoscopy (patient-related contraindications to cytoreductive surgery or prediction of suboptimal cytoreduction based on preoperative imaging), the procedure was performed according to the protocol proposed by Fagotti et al. [28], and all predefined localizations and their status were noted and recorded. The presence or absence of the disease was assessed in the anatomic areas listed in Table 1. Metastatic disease was recorded as being present or absent; the diameters of the lesions or other size quantification was not done.

Decisions concerning the management of patients with EOC were made by the oncology council at our center. Patients in poor general condition (Eastern Cooperative Oncology Group 3 or 4) with severe co-morbidities, multiple metastases in the liver parenchyma, lung metastasis, or enlarged supraclavicular/mediastinal lymph nodes were classified as inoperable and excluded from the study. Histopathological confirmation was obtained by ultrasound-guided biopsy or paracentesis, and patients were referred for chemotherapy. Patients with suspicion of infiltration of the vessels of the liver hilum or mesentery of the small intestine and carcinomatosis covering a large area of the bowel serosa were referred for a diagnostic laparoscopy to visually inspect all the anatomical areas evaluated during the ultrasound examination of this study.

The FIGO system was used for staging EOC [27]. To simplify the categorization and calculations, we divided EOC stages into two entities: early (FIGO I–IIA) and advanced (FIGO IIB–IVB). Surgical complexity was calculated as described elsewhere [26]. Complete cytoreduction (R0) was defined as no macroscopic residual disease after completion of surgery. Optimal cytoreduction (R < 1 cm) was defined as macroscopic residual disease of <1 cm diameter for each lesion. Suboptimal cytoreduction (R > 1 cm) was defined as macroscopic residual disease ≥ 1 cm.

Systematic lymphadenectomy was performed in macroscopically early EOC. In patients who underwent cytoreductive surgery, regarding lymphadenectomy, only enlarged suspected lymph nodes were removed. Resection of liver metastases was performed in cases of isolated parenchymal lesions.

The reference standard (surgical and histology) results were not available to the ultrasound examiners. Detailed information of the index tests, except for the general clinical diagnosis about suspected adnexal mass or suspicion of primary peritoneal cancer, were not available to the assessors of the reference standard.

### 2.5. Statistical Analysis

Statistical analysis was performed using R [29]. Sensitivity, specificity, positive predictive value (PPV), negative predictive value (NPV), and overall accuracy were calculated. Data for each factor are presented as a contingency table. Sensitivity was measured by the ratio of true positive cases (TP) to all cases with an actual positive condition (i.e., TP and false negatives (FN)), which was calculated by TP/(TP + FN). Specificity was defined as the ratio of true negative cases (TN) to all negative cases (false positive (FP) and TN), which was calculated by TN/(FP + TN). The PPV was calculated using the formula TP/(TP + FP), and NPV was calculated using the formula TN/(FN + TN). Overall accuracy was calculated using the formula (TP + TN)/(TP + FP + TN + FN). Differences between groups (no/yes, high/low) were analyzed using Fisher’s exact and chi-square tests. For both tests, statistical significance was set at an alpha of <0.05.

The analysis of ultrasound performance for predicting surgical complexity [26] and residual disease was analyzed only if open surgery with attempted cytoreduction for advanced disease or full staging for the early-stage disease were performed (cases who underwent diagnostic laparoscopy were excluded). The statistical calculation for surgical complexity was performed using two different levels: low/intermediate and high. For the statistical analysis of this study, we only used two groups: the R0 and no-R0 groups (R < 1 cm and R > 1 cm).

This study used the Standards for Reporting Diagnostic Accuracy guidelines [30]. 

There were no indeterminate index test or reference standard results. 

Participants with missing ultrasound examinations or those who were not undergoing surgery were excluded from the study.

We aimed to conduct variability analysis on the diagnostic accuracy of the ultrasound depending on body mass index (BMI) and the presence of ascites. 

During the planning process of the study, we carried out two sample size calculations. The first analysis was performed to assess the specificity (and the sensitivity). Assuming that all patients had EOC and 70% of them had the advanced disease (lesions in the abdominal cavity and lesser pelvis that can be detected by diagnostic imaging examination), a minimum of 103 patients (including 72 with advanced disease) were required to observe a percentage specificity increase from 0.70 (minimal clinical usefulness; null hypothesis (H0)) to 0.90 (desirable value and clinically useful; alternative hypothesis (H1)), with >80% power (the probability of type II error) and a statistical significance of *p* < 0.05 (the probability of type I error). This sample size was also sufficient for detecting an increase in sensitivity values from 0.70 to 0.90 (N = 44) [31]. The second analysis was performed to evaluate the accuracy of the ultrasound examination (by calculating the area under the receiver operating curve (AUC)). Assuming the null hypothesis of 0.65 AUC (unsatisfactory test accuracy for clinical practice) and the alternative hypothesis of ≥0.80 AUC (acceptable accuracy for clinical practice; i.e., δ = 0.15, with a 95% confidence interval and 80% test power), and assuming that 70% of patients have advanced disease and 30% have an early-stage disease, the total size of the study group needed to be at least 117 [32].

## 3. Results

**Figure 14:**
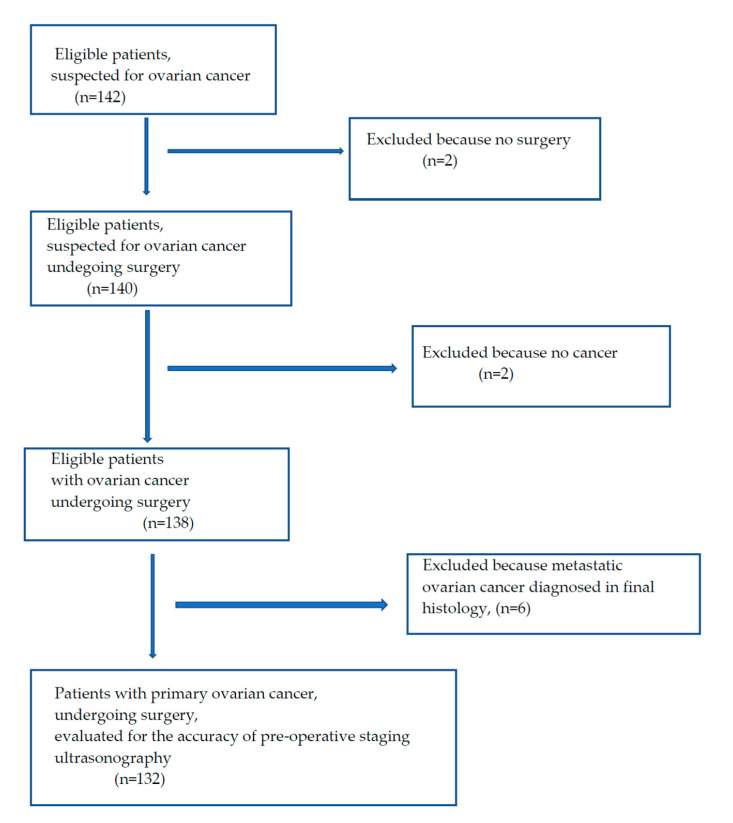
Patient flowchart.

We enrolled 142 patients in the study. Ten women were excluded for various reasons, and 132 were included in the final sample. Detailed data are presented in Figure 14.

The average time between ultrasound examination and surgery was 5 days.

Patient clinical characteristics are presented in Table 2.

There were 113 patients who underwent open surgery. Those with macroscopically early disease (stages I–IIA) represented 10.5% of the study population, and all underwent open surgical staging: longitudinal incision from the symphysis pubis to xiphoid process, systematic inspection of all abdominal cavity and retroperitoneal space, hysterectomy, bilateral oophorectomy, total omentectomy (infra-stomach), and systematic pelvic and paraaortic lymphadenectomy, up to the level of the left renal vein. Patients with advanced disease, who were fit enough to undergo surgery, and in whom we did not suspect unresectable disease (e.g., small bowel mesentery root involvement), underwent debulking surgery. Whenever feasible and necessary to obtain complete macroscopic cytoreduction the following procedures were performed: modified posterior exenteration (radical oophorectomy) with anastomosis, pelvic and abdominal wall peritonectomy, right and left diaphragm peritoneum stripping, splenectomy (if necessary with distal pancreatectomy), resection of isolated parenchymal liver lesions, lesser omentum resection, peritonectomy in the liver hilum and pouch of Morison, partial small bowel resections, hemicolectomy, the opening of the right pleural cavity and resection of pleural implants on the diaphragm part, resection of enlarged cardiophrenic lymph nodes, resection of enlarged retroperitoneal lymph nodes in the pelvis and paraaortic region.

Ultrasonography findings are shown in Table 3. The mean time of ultrasound examination was 30 min (range: 15–40).

The performance of ultrasonography for the detection of lesions in the abdominal cavity and pelvis, as well as the prediction of disease stage, surgical complexity, and residual disease, are shown in Table 4.

The performance of ultrasonography for staging and prediction of surgical outcome, stratified by BMI value and ascites presence (as detected by ultrasound), is presented in Tables S1 and S2. There were no major clinically significant differences in ultrasound performance depending on BMI. However, specificity for frozen pelvis was higher in patients with BMI ≤ 30 as compared with that in obese patients. Comparison of ultrasound performance stratified by the presence or absence of ascites revealed that predictions were significantly better for negative findings in the pelvis and abdominal wall peritoneum, spleen hilum, and rectum if ascites were detected in the ultrasound compared with those who had no ascites in the ultrasound. Interestingly, performance of ultrasonography was better for predicting right diaphragm involvement in patients without ascites than for those with ascites. There were no other clinically meaningful differences in the assessment of disease status or predictions based on ultrasonography between those with and without ascites on the ultrasound.

## 4. Discussion

We demonstrated that ultrasonography performed by a gynecological oncologist can be highly accurate for planning treatment for patients with EOC. We found that ultrasonography is highly accurate not only in detecting disease in various anatomical areas, including crucial regions such as the small bowel mesentery root, but also in predicting disease stage, surgical complexity, and residual disease.

Detection of omentum involvement with ultrasonography is technically easy and has high sensitivity and specificity parameters in both our and other studies [12,14,16]. However, detection of omentum involvement alone does not provide useful information for clinical practice because, even if involved with tumors, omentectomy is a simple procedure that is performed frequently by every gynecological oncologist. Testa et al. [12] noted that omentum infiltration may be considered a marker for risk of incomplete cytoreduction. From a practical perspective, it may be more useful if one could distinguish between separate omentum involvement versus cancerous omentum infiltration on the transverse colon or small bowel because it would imply higher surgical complexity, longer operative time, and generally more complicated procedures during the surgery. However, our study was not designed to address this issue. Therefore, future studies should aim to stratify ultrasonography of omentum involvement into omentum only versus omentum plus bowel. 

Small bowel mesentery root involvement is of great clinical importance because once this condition is detected at surgery, achieving complete cytoreduction is generally unfeasible. This issue is crucial, even when laparoscopic evaluation is undertaken before surgery, and the Fagotti score should not be calculated if the small bowel mesentery root is involved [28]. Previous studies on ultrasound accuracy on this issue have reported disappointing sensitivity (23.5–66.7%) and specificity (87.8–100%) [12,14,16]. We were able to detect small bowel mesentery root involvement with a sensitivity of 95.5%, and correct diagnoses of no involvement were achieved in 92.3% of cases. 

Detection of peritoneum involvement in the abdominal cavity and pelvis is important in clinical practice because peritonectomy is an additional procedure that causes prolongation of operation time. Transvaginal ultrasound for the assessment of pelvic peritoneum status has a sensitivity of 75–85% and a specificity of 94–97% [14,16,33]. Performance of ultrasound assessment of the abdominal peritoneum has been reported with a sensitivity of 34–91.5% and a specificity of 91–99% [12,14,16]. In our study, sensitivity and specificity for pelvic peritoneum involvement were 80% and 81%, respectively, and for abdominal wall peritoneum involvement, they were 88% and 53%, respectively. We could predict negative findings in the pelvis and abdominal wall peritoneum significantly better if ascites were detected in the ultrasound compared with when no ascites were detected in the ultrasound. 

Ascites are the most common symptom of advanced EOC. In general, it is easily diagnosed by ultrasound, as shown previously [12,16] and in our study. The presence of free fluid facilitates the assessment of intraabdominal organs. It can be exploited as a specific radiological contrast or window because it allows for a more accurate assessment of the parietal peritoneum, possible changes on the surface of the intestines (carcinomatosis), and mesenteric infiltration of the small intestine.

Liver and spleen parenchymal lesions could be preoperatively diagnosed with > 95% sensitivity and specificity. However, note that in a small group of patients with suspected lesions in the liver and spleen parenchyma in the ultrasound examination, verification of negative imaging findings with full histopathological evaluation of the liver and spleen parenchyma was not possible (splenectomies were performed mostly for spleen hilum involvement, and only the palpable and visualizable liver parenchymal lesions were resected, if resection was feasible). Moreover, a small number of patients with positive findings in the liver and spleen parenchyma have been reported in other studies [12,16]. In the future, it would be worth conducting a study in a larger group of patients with suspected lesions in the liver parenchyma, which would allow for more objective results. In addition, such studies should comprise patients with recurrent EOC because, in primary diagnosis and treatment settings, parenchymal liver lesions are rather a marker for non-ovarian malignancy, as described earlier [34]. 

Localization of liver hilum involvement is important when planning the treatment of patients with advanced EOC. Cancerous involvement of the portal vein, hepatic artery, or bile duct renders complete cytoreduction impossible, whereas resection of the involved peritoneum covering these vessels requires great surgical skills and experience and is associated with a high risk of intra- and postoperative complications. Therefore, accurate liver hilum assessment is important for gynecological oncologists. In our study, the overall accuracy was 91% for the assessment of this localization, which suggests that ultrasound could be considered a standard procedure for the assessment of this localization in patients with EOC. However, results should be interpreted with caution because others have reported high specificity (100%), but very low sensitivity for detection of lesions in this area [16]. 

The detection of spleen hilum involvement is important for planning the extent and time of surgery, and prophylactic vaccination before treatment may also be considered. However, it is not a crucial issue because, even if it is not suspected during the pre-operative workup and is found during surgery, splenectomies can be performed by most trained gynecological oncologists. Nevertheless, suspecting spleen hilum involvement in ultrasonography, one could suggest preoperative prophylactic vaccination if a splenectomy is planned.

Diaphragm peritoneum imaging with ultrasound is not an easy procedure, mainly because the ribs can hinder the ultrasound examination. The sensitivity and specificity of diaphragm examination were 30.8% and 98.9%, respectively, in a study by Fischerova et al. [14]. In contrast, we revealed a sensitivity of 90% and 95% for the right and left diaphragms, respectively. However, achieving correct assignment of negative findings in the diaphragm with ultrasound was problematic, with a specificity of 21–60%. Unless there is no significant involvement of the diaphragm region (e.g., localization next to the upper hepatic veins or large area full-thickness infiltration), the presence of small/miliary nodules on the diaphragm peritoneum is not an issue for most gynecological oncologists, and resection of the peritoneum can be performed easily. Nevertheless, it is clinically useful to know the status of this localization because it would complement other information for planning adequate management of each patient. Insights regarding ultrasonography applications for diaphragm imaging of both the abdominal and pleural sides have been presented elsewhere [35]. 

The frozen pelvis is a condition whereby all intraperitoneal pelvic organs form cancerous adhesions with each other. Assessing ultrasonography is relatively easy owing to the use of a vaginal transducer and dynamic examination. Accurate assessment of the pelvic structures and finding neoplastic changes involving the uterus, ovaries, rectum, and pelvic peritoneum in the form of an immobile tumor implies higher surgical complexity and longer procedure duration. In our research, we found 94% sensitivity and 76% specificity of ultrasonography for assessing this parameter. Others have reported a sensitivity of 94% and a specificity of 97% for the prediction of the frozen pelvis [12].

The rectosigmoid colon is frequently involved alongside other intraperitoneal pelvic organs in patients with advanced EOC. Fortunately, most gynecological oncologists are trained in surgical techniques, which enable complete cytoreduction in this area, such as by performing modified posterior exenteration. Nevertheless, good quality presurgical imaging is valuable because suspected rectal infiltration or frozen pelvis affects surgical complexity. Ultrasound presentations of normal rectum layers and involvement of the rectum have been described previously [15]. Earlier studies have shown 59–86% sensitivity and 95–99% specificity for detection of rectosigmoid infiltration using transvaginal ultrasound [12,14,15,16]. Moreover, precise information on the degree of rectosigmoid infiltration (superficial or deep) can be obtained [14], or massive pelvic involvement can be predicted [12]. We achieved comparable results (sensitivity of 81% and specificity of 92%) of ultrasound performance in this area.

We also assessed the stage of disease based on ultrasound imaging. The accuracy of ultrasound prediction of disease stage was 86% and it did not depend on the presence of ascites or BMI value.

Surgical complexity can be calculated based on imaging and presented in three levels, as previously described [26]. To the best of our knowledge, we are the first to report this parameter based on pre-operative ultrasonography. We showed high accuracy in predicting the level of this parameter, regardless of the patient’s BMI or presence of ascites. Although most experienced gynecological oncologists perform numerous surgical procedures (e.g., intestinal resections, splenectomies, and peritonectomies), in cases of substantial involvement of many sites in the abdominal cavity, the number of procedures accumulates, and the time of the operation is prolonged significantly. Therefore, during the process of qualifying the patient for surgery, careful assessment of the extent of the surgery, the patient’s condition, co-morbidities, and possible complications is necessary.

The prediction of achieving macroscopically complete or incomplete cytoreduction is of greatest importance because it impacts the management plan for PDS or neoadjuvant chemotherapy and interval debulking surgery. Residual disease prediction assessed by ultrasound was reasonably accurate (overall accuracy of 80.5%) in our study. However, we used subjective assessments based on comprehensive examinations. The ultrasound scoring system developed by Testa et al. [12] may be more practical and objective for predicting residual disease. Although, care should be taken when using one tool or score to predict the outcome of surgery. In fact, one method should not be relied upon because there are too many variables (clinical, tumor biology, and imaging) that need to be considered together when planning complex treatments of patients with advanced EOC. 

Initially, we planned to perform an analysis of retroperitoneal lymph nodes involvement. However, because not all patients had these regions surgically explored and histologically examined, this parameter was not analyzed. Nevertheless, the retroperitoneal space should be scanned to search for enlarged lymph nodes that could be metastatic and should be resected during the surgery. Moreover, if one relies on a laparoscopic evaluation only before treatment, then theoretically, ultrasonography should be regarded as an additional complementary tool to laparoscopy because the retroperitoneal space can be scanned using ultrasound but not with diagnostic laparoscopy (in the way defined by Fagotti et al. [36]). Future research on the issue of ultrasound accuracy for retroperitoneal space pretreatment staging should focus on patients with macroscopically early-stage disease (FIGO I–IIA) who have undergone systematic paraaortic and pelvic lymphadenectomy for staging purposes. One could argue that ultrasound is redundant because standard CT provides information on the status of the disease in the abdominal cavity and retroperitoneal space. However, as described in a recent study, CT imaging shows a limited diagnostic performance for the detection of lymph node metastases in patients with EOC, with a sensitivity of 40.7%, a specificity of 89.1%, a PPV of 80.0%, and an NPV of 58.3% [37].

Our study was an observational study in patients with EOC who were not selected, who underwent ultrasound imaging and surgery performed by a gynecological oncologist in a department in a tertiary oncology center, that is certified by ESGO for the surgical treatment of patients with advanced ovarian cancer. Moreover, physicians performing ultrasonography underwent regular and additional training in sonology. Thus, the results of our study represent this specific clinical setting and cannot be generalized and simply copied into general gynecological, medical oncology departments, or even in gynecologic oncological units, where physicians have less experience in sonology and surgery. 

In the studies performed to date, the limitations of ultrasound examination in EOC include the assessment of infiltration of the root of bowel mesentery. We believe that a detailed ultrasound assessment, as well as the use of dynamic ultrasound, allowed us to assess the mesentery of the small intestine with great accuracy. The examination is more difficult in patients without ascites because there is no space between the organs, bowel, and abdominal/pelvic wall. For ambiguous imaging results, we believe that laparoscopic assessment of the abdominal cavity before planned cytoreductive surgery is an ideal tool to complement imaging diagnostics and allows for a well-planned treatment of patients with advanced EOC.

Another limitation of ultrasonography (and other imaging methods) is the detection of miliary carcinomatosis. Probably, staging laparoscopy is the most accurate method for this issue. However, miliary carcinomatosis has no major clinical significance for the gynecological oncologist, unless present on the bowel serosa. In the case of superficial, small peritoneal implants (e.g., on the abdominal wall, peritoneum on small bowel mesentery, Glisson’s capsule), resection is feasible with partial or total peritonectomy. Because of these observations and practice, we did not measure the lesions of metastatic disease. 

Some anatomical areas were affected by cancer in only a few patients (e.g., the liver and spleen parenchyma). Moreover, one can imagine the situations where a focal deep parenchymal lesion is not detected in ultrasonography, it is also not seen or palpable during surgery. Furthermore, a histological report of the whole liver cannot be obtained, unless by autopsy. Thus, the statistical results of such parameters must be interpreted with caution. Further studies in larger groups of patients are needed. Another limitation is the ultrasound evaluation of extra-abdominal disease. Assessments of thoracic disease, mediastinal lymph metastases, or brain metastases require CT, although assessment of lower parts of pleural cavities performed using ultrasound with acceptable diagnostic accuracy was recently described [35].

## 5. Conclusions

Ultrasonography performed by a gynecological oncologist can be a precise imaging tool for the assessment of the intra-abdominal spread of ovarian cancer; moreover, it is easily accessible, economical, quick, and does not overburden the patient. During a pretreatment workup, a gynecological oncologist with experience in the operating room and ultrasonography, using the knowledge of ovarian cancer spread characteristics, can accurately detect lesions in the abdominal cavity and pelvis, recognize critical disease manifestations (bowel mesentery root), and predict disease stage, surgical complexity, and residual disease, which allows the accurate qualification of patients for PDS or neoadjuvant chemotherapy. Future studies should focus on the usage of technical developments in the field of ultrasound machines, improvements in contrast-enhanced ultrasonography, and continuous multidisciplinary education in gynecological oncology ultrasonography.

## Figures and Tables

**Figure 1:**
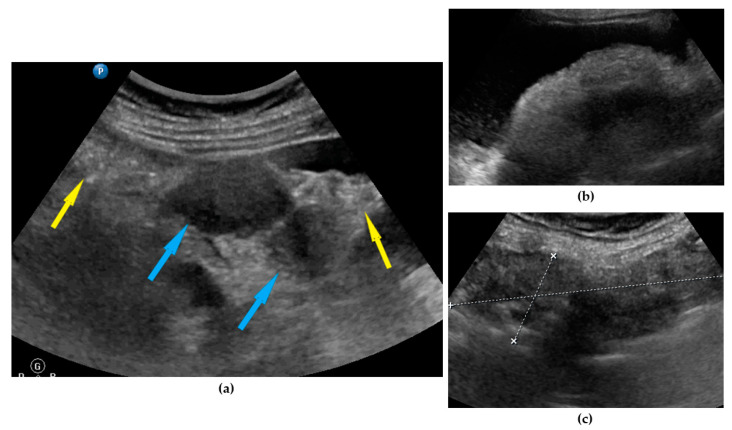
Ultrasound images of omentum involvement: (**a**) focal infiltration (blue arrows identify tumors, yellow arrows identify normal omentum); (**b**) diffuse omental infiltration with ascites; (**c**) diffuse omental infiltration without ascites.

**Figure 2:**
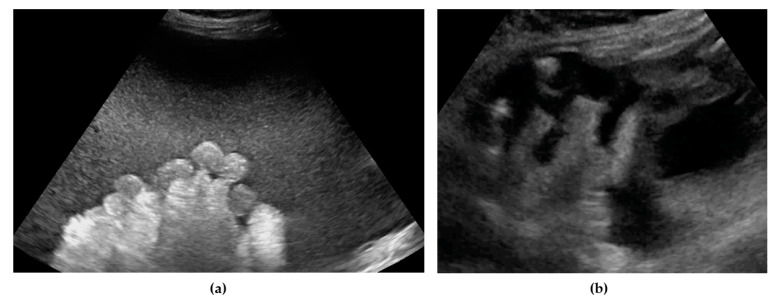
Ultrasound images of small bowel mesentery: (**a**) involvement of mesentery root (mesenteries adhere to each other); (**b**) no involvement of mesentery root (separation of mesenteries). Note: the best visualization in dynamic examination (see Video S2).

**Figure 3:**
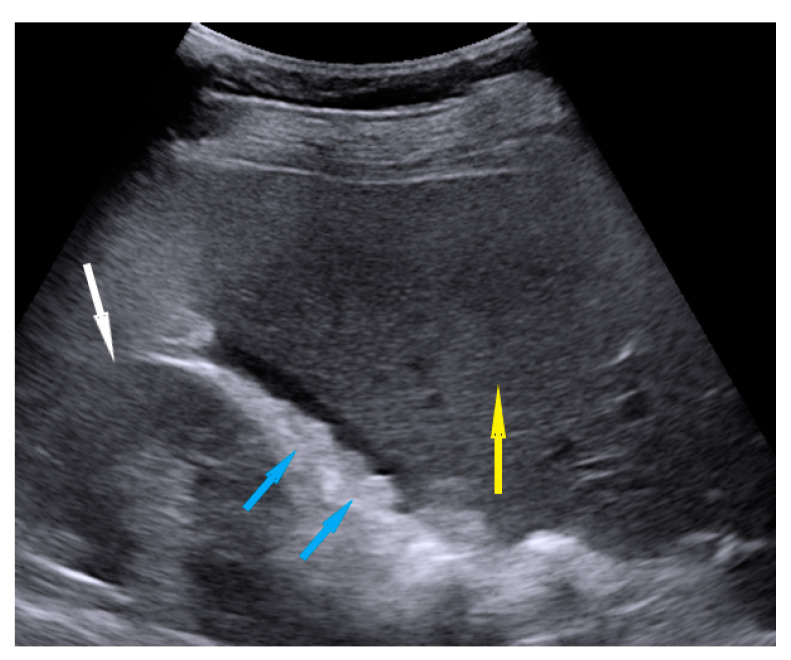
Ultrasound image of nodules on the peritoneum of Morison pouch. Blue arrows identify nodules, yellow—liver, white—kidney.

**Figure 4:**
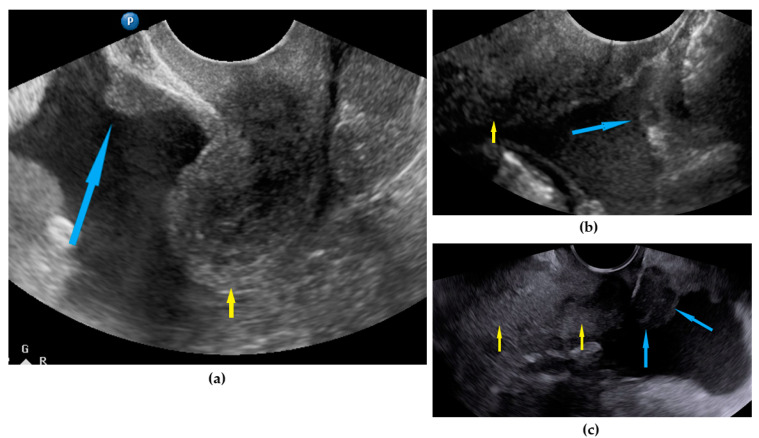
Ultrasound images of pelvic peritoneum involvement: (**a**) nodule on bladder peritoneum; nodule (**b**) and tumor (**c**) on pouch of Douglas peritoneum. Arrows: blue identify nodules, yellow identify uterus.

**Figure 5:**
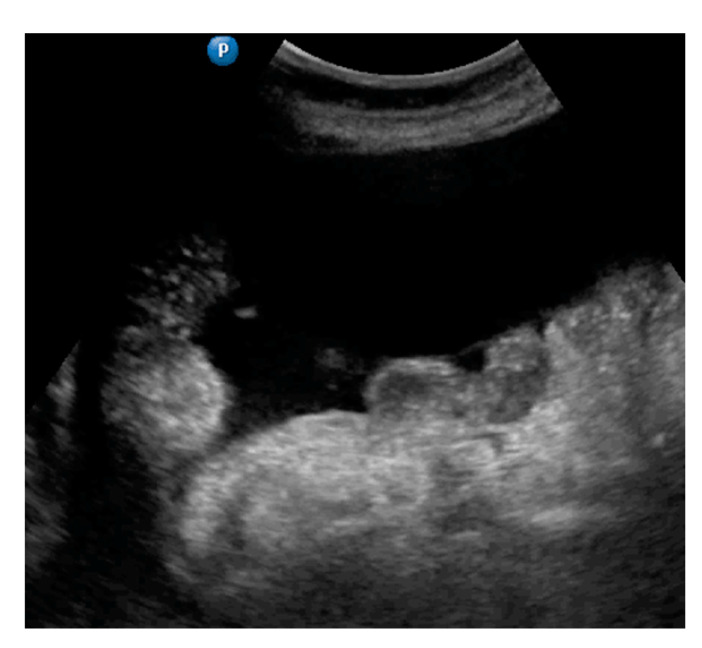
Ultrasound image of ascites.

**Figure 6:**
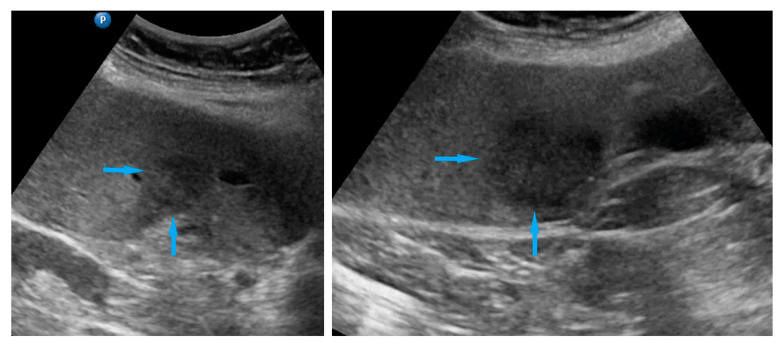
Ultrasound images of hepatic parenchymal lesions. Blue arrows identify tumors.

**Figure 7:**
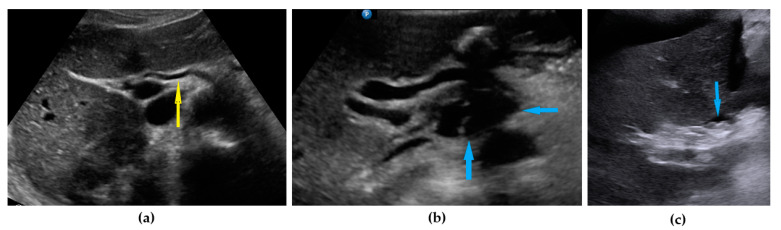
Ultrasound images of hepatic hilum: (**a**) normal hilum (yellow arrow identify hepatic artery); (**b**) tumor under hepatic artery; (**c**) carcinomatosis on the peritoneum around hepatic hilum. Blue arrows identify tumors.

**Figure 8:**
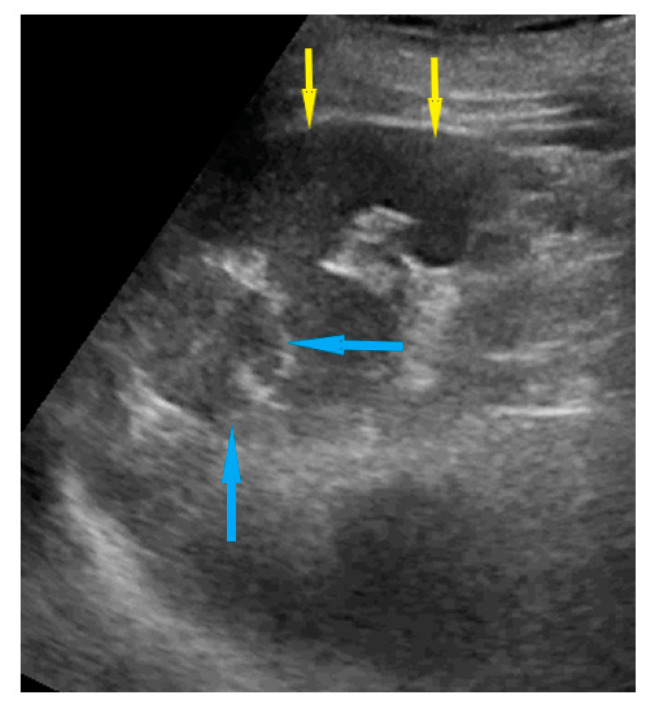
Ultrasound image of spleen parenchymal lesions (blue arrows) (yellow arrows identify normal spleen parenchyma).

**Figure 9:**
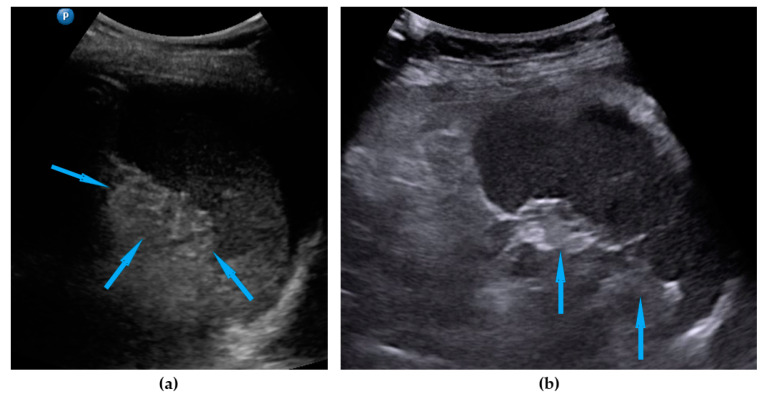
Ultrasound images of spleen hilum involvement: (**a**) diffuse infiltration; (**b**) focal lesions. Blue arrows identify lesions.

**Figure 10:**
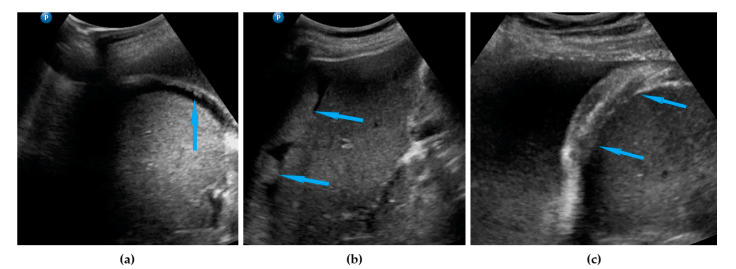
Ultrasound images of right diaphragm peritoneum involvement: (**a**) nodules; (**b**) tumors; (**c**) diaphragm thickening. Blue arrows identify lesions.

**Figure 11:**
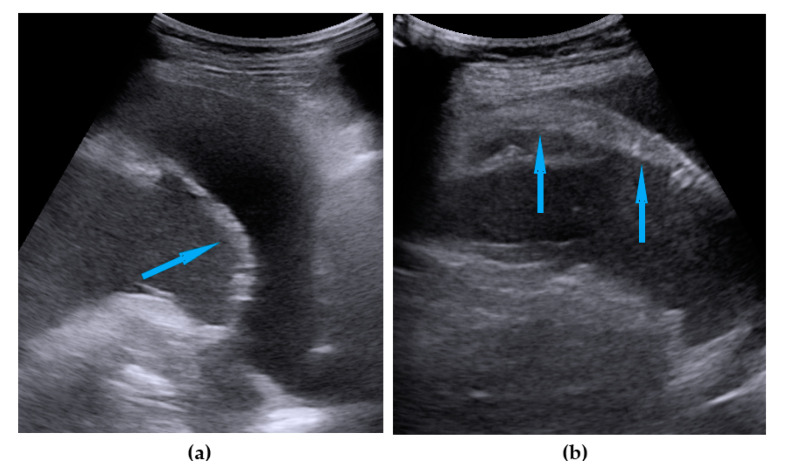
Ultrasound images of left diaphragm peritoneum involvement: (**a**) nodules; (**b**) diaphragm thickening. Blue arrows identify lesions.

**Figure 12:**
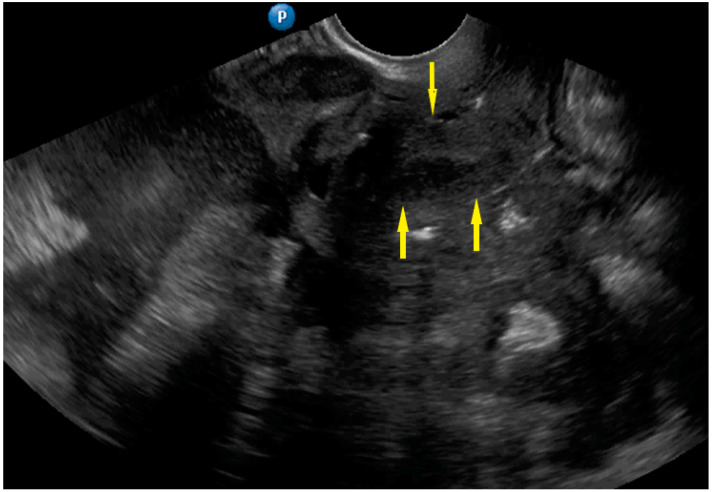
Ultrasound image of frozen pelvis. Yellow arrows identify uterus. There is involvement of bladder peritoneum and massive involvement of the compartment posterior to the uterus.

**Figure 13:**
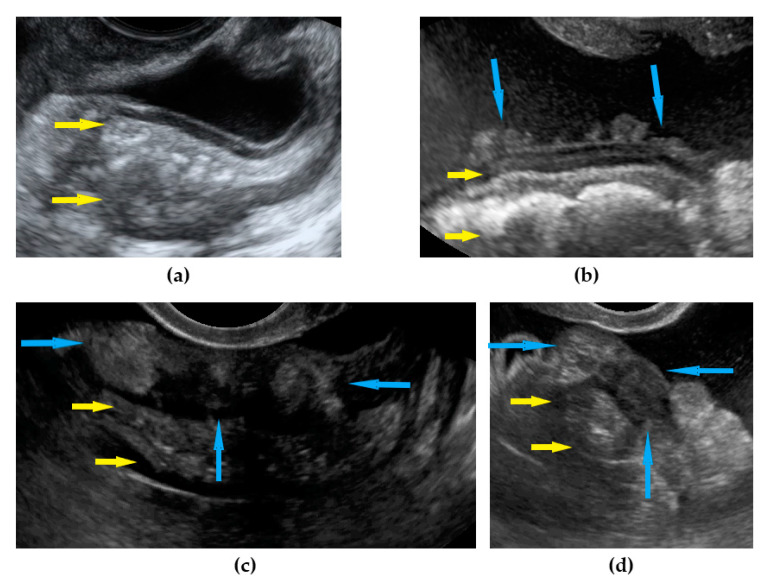
Ultrasound images of rectum: (**a**) normal rectum; (**b**) nodular carcinomatosis on the peritoneal surface of the rectum; (**c**) sheet-like involvement of the rectum wall (longitudinal view); (**d**) sheet-like involvement of the rectum wall (transverse view). Yellow arrows identify rectum, blue arrows identify lesions.

**Table 1:** Anatomical areas and parameters evaluated using ultrasonography. Criteria and definitions for considering localization, involvement, and parameters.

Parameter	Definition	Figure	Video
Omentum	Focal infiltration of omentum: hypoechogenic nodules with discrete vascularization. Diffuse infiltration: omental cake appears as a nodular, perfuse, and non-peristaltic tumor that is located between the anterior abdominal wall and bowel loops.	Figure 1	Video S1
Small bowel mesentery root	Involvement is suspected when bowel loops have poor mobility and are “fixed together” in the dynamic ultrasound examination with a cauliflower-like image.	Figure 2	Video S2
Peritoneum, abdomen	Abdomen carcinomatosis manifest as hypoechogenic lesions over the peritoneal surface of the paracollic gutters or internal abdominal wall.	Figure 3	Video S3
Peritoneum, pelvis	Pelvic carcinomatosis manifests as hypoechogenic lesions over the peritoneal surface of the pelvic wall: laterally, in the pouch of Douglas (no rectum involvement) or the bladder in the uterine serosa.	Figure 4	Video S4
Ascites	Fluid outside the pouch of Douglas, recorded as being present or absent ^1^	Figure 5	Video S5
Liver, parenchymal lesions	Single or multiple focal parenchymal lesions (with a “halo“ sign, necrosis, and indistinct borders) in the liver.	Figure 6	Video S6
Liver hilum	Presence of nodules or rigid structures in the region of the hepatic hilum.	Figure 7	Video S7
Spleen, parenchymal lesions	Single or multiple focal parenchymal lesions (with a “halo“ sign, necrosis, and indistinct borders) in the spleen.	Figure 8	Video S8
Spleen, hilum	Presence of nodules or rigid structures in the region of the spleen hilum.	Figure 9	Video S9
Diaphragm, right	Carcinomatosis manifests as hypoechogenic lesions over the peritoneal surface of the right diaphragm.	Figure 10	Video S10
Diaphragm, left	Carcinomatosis manifests as hypoechogenic lesions over the peritoneal surface of the left diaphragm.	Figure 11	Video S11
Frozen pelvis	Massive pelvic involvement: hypoechogenic tissue in the peritoneum in the pouch of Douglas, forming cohesion between ovarian masses, bowel, uterus, and posterior pelvic wall. It manifests in the dynamic ultrasound examination: absence of sliding sign between the rectum and uterus/ovaries and between the uterus, urinary bladder, and pelvic walls.	Figure 12	Video S12
Rectum-sigmoid	Suspected involvement of the rectosigmoid wall manifests as the presence of metastases over the wall of the rectum or sigmoid.	Figure 13	Video S13
Stage prediction	Subjective prediction of disease stage: early (FIGO IA–IIA) or advanced (FIGO IIB–IVB).	-	-
Surgical complexity prediction	Subjective prediction of surgical complexity, defined as described elsewhere ^2^, if open surgery with cytoreduction attempted or full staging.	-	-
Residual disease prediction	Subjective prediction of residual disease after surgery if debulking surgery is attempted. Note: residual disease prediction based on ultrasonography was made without stratification into advanced or early disease.	-	-

^1^ Ascites definition from Timmerman D. et al. [25]. ^2^ Surgical complexity as defined in publication Aletti G.D. et al. [26]. Note: metastatic disease in all localizations was recorded as being present or absent; the diameters of the lesions or other size quantification was not done.

**Table 2:** Patient clinical characteristics.

Parameter	Data
Age, years, median (range)	62 (32–82)
BMI ^1^, kg/m^2^, median (range)	26.8 (15.8–38.4)
CA125 ^2^, U/mL, median (range)	454 (20–20,050)
D-dimer, ng/mL, median (range)	3416 (160–25,000)
ASA ^3^ score, *n* (%)	
I	2 (1.5%)
II	108 (82.4%)
III	21 (16.1%)
Performance status ^4^	
0	23 (17.6%)
1	81 (61.8%)
2	23 (17.6%)
3	4 (3.0%)
FIGO ^5^ stage	
I	13 (9.8%)
IIA	1 (0.7%)
IIB	10 (7.5%)
IIIA1	8 (6.0%)
IIIB	12 (9.0%)
IIIC	64 (48.5%)
IVA	9 (6.8%)
IVB	15 (11.7)
Histology, type	
Serous	95 (71.9%)
Endometrioid	13 (9.8%)
Clear Cell	7 (5.3%)
Mucinous	7 (5.3%)
Non-differentiated	3 (2.4%)
Mixed	7 (5.3%)
Histology, grade	
G1	6 (4.5%)
G2	6 (4.5%)
G3	120 (91.0%)
Surgery	
Open (PDS ^6^/IDS ^7^ or full staging)	113 (85.6%)
Diagnostic laparoscopy	19 (14.4%)
Surgical complexity ^8^	
for open surgery, FIGO ^5^ IIB-IVB	
low	16 (16%)
intermediate	41 (41%)
high	43 (43%)
Residual disease	
for open surgery, FIGO ^5^ IIB-IVB	
R0 (complete macroscopic resection)	66 (66%)
R < 1 cm	7 (7%)
R > 1 cm	27 (27%)

^1^ BMI, body mass index; ^2^ CA125, cancer antigen 125; ^3^ ASA, American Society of Anesthesiologists Philical Status Classification System; ^4^ Performance status (PS) according to Eastern Cooperative Oncology Group; ^5^ FIGO, International Federation of Gynecologists and Obstetricians; ^6^ PDS, primary debulking surgery; ^7^ IDS, interval debulking surgery (after adjuvant chemotherapy); ^8^ Surgical complexity as defined in publication Aletti D.G. et al. [26].

**Table 3:** Localizations of lesions as detected by ultrasonography, and ultrasound predictions of disease stage, surgical complexity, and residual disease.

Parameter	Ultrasound
Omentum	
No involvement	56 (42.4%)
Nodules	7 (5.3%)
Gross involvement	69 (52.3%)
Small bowel mesentery root involvement, *n* (%)	17 (12.9%)
Peritoneum, abdomen, lesions, *n* (%)	46 (34.8%)
Peritoneum, pelvis, lesions, *n* (%)	88 (66.7%)
Ascites, yes, *n* (%)	65 (49.2%)
Liver, parenchymal lesions detected, *n* (%)	4 (3.0%)
Liver hilum involvement, *n* (%)	9 (6.8%)
Spleen, parenchymal lesions detected, *n* (%)	1 (0.7%)
Spleen, hilum involvement, *n* (%)	39 (29.5%)
Diaphragm, right, lesions, *n* (%)	52 (39.4%)
Diaphragm, left, lesions, *n* (%)	12 (9.0%)
Frozen pelvis detected, *n* (%)	31 (23.5%)
Rectum-sigmoid involvement, *n* (%)	70 (53.0%)
Stage prediction ^1^	
early	31 (23.5%)
advanced	101 (76.5%)
Surgical complexity prediction ^2^	
low	1 (0.7%)
intermediate	58 (43.9%)
high	73 (55.4%)
Residual disease prediction ^3^	
R0 (no macroscopic disease)	98 (74.9%)
R < 1 cm	9 (6.8%)
R > 1 cm	25 (18.3%)

^1^ stage prediction based on ultrasonography: early or advanced; ^2^ Surgical complexity as defined in publication Aletti D.G. et al. [26], prediction based on ultrasonography; ^3^ Residual disease after surgery—prediction based on ultrasonography, without stratification into early or advanced disease.

**Table 4:** Accuracy of ultrasonography in the defined localizations and predictions of disease stage, surgical complexity, and residual disease.

Parameter	Sensitivity %	Specificity %	PPV ^1^ %	NPV ^2^ %	Accuracy %	*p*
Omentum, gross involvement	96.9	89.4	89.9	96.7	93.1	<0.001
Omentum, small nodules	17.4	97.2	57.1	84.6	83.1	
Small bowel mesentery root	95.5	92.3	99.1	70.6	95.2	<0.001
Peritoneum, abdomen	87.7	53.4	59.5	84.8	68.5	<0.001
Peritoneum, pelvis	80.0	81.2	55.8	93.2	80.9	<0.001
Ascites	95.5	96.9	97.0	95.4	96.2	<0.001
Liver, parenchymal	99.2	100	100	75.0	99.2	<0.001
Liver hilum	99.1	42.1	91.1	88.9	90.9	<0.001
Spleen, parenchymal ^3^	100	100	100	100	100	0.008
Spleen, hilum	90.3	76.9	90.3	76.9	86.4	<0.001
Diaphragm, right	90.0	59.5	58.4	90.4	71.3	<0.001
Diaphragm, left	94.8	20.6	77.1	58.3	75.4	0.01
Frozen pelvis	94.8	76.5	91.9	83.9	90.0	<0.001
Rectum	81.4	91.9	91.9	81.4	86.4	<0.001
Stage prediction ^4^	88.0	75.0	94.1	58.1	85.6	<0.001
Surgical complexity prediction ^5^	83.7	72.9	65.5	87.9	77.0	<0.001
Residual disease prediction ^6^	41.2	97.5	87.5	79.4	80.5	<0.001

^1^ PPV, positive predictive value; ^2^ NPV, negative predictive value; ^3^ note, that parenchymal metastases were detected with ultrasonography and confirmed in histology in a single patient; ^4^ stage prediction: early or advanced; ^5^ Surgical complexity as defined in publication Aletti D.G. et al. [26], analyzed only if open surgery with attempted cytoreduction or full staging (*n* = 113), stratified into two subgroups: low/intermediate and high; ^6^ Residual disease after surgery—prediction based on ultrasonography, without stratification into early or advanced disease, analyzed only if open surgery with attempted cytoreduction or full staging (*n* = 113).

## Data Availability

The study protocol can be accessed on demand of interested researcher if justified.

## Supplementary Materials

The following are available online at https://zenodo.org/record/5482666#.YUWtOH0RWUl, Table S1. Accuracy of ultrasonography for the detection of lesions in different localizations and predictions of disease stage, surgical complexity, and residual disease, stratified by patients’ BMI. Table S2. Accuracy of ultrasonography for the detection of lesions in different localizations and predictions of disease stage, surgical complexity, and residual disease, stratified by presence of ascites detected by ultrasonography. Video S1: Ultrasound video of omental involvement. Video S2: Ultrasound and laparoscopic video of small bowel mesentery root involvement. Video S3: Ultrasound video of carcinomatosis on peritoneum of Morison’s pouch. Video S4: Ultrasound video of pelvis peritoneum involvement. Video S5: Ultrasound video of ascites. Video S6: Ultrasound video of parenchymal lesions in the liver. Video S7: Ultrasound and laparoscopic video of hepatic hilum involvement. Video S8: Ultrasound video of parenchymal lesions in the spleen. Video S9: Ultrasound video of spleen hilum involvement. Video S10: Ultrasound video of the right diaphragm peritoneum involvement. Video S11: Ultrasound video of the left diaphragm peritoneum involvement. Video S12: Ultrasound and laparoscopic video of frozen pelvis. Video S13: Ultrasound video of rectum involvement.

## Abbreviations

CT	computed tomography
EFSUMB	European Federation of Societes for Ultrasound in Medicine and Biology
EOC	epithelial ovarian cancer
ESGO	European Society of Gynecological Oncology
F-FDG PET	18F-fluorodeoxyglucose positron emission tomography
FIGO	International Federation of Gynecology and Obstetrics
FN	false negative
MI	mutual information
MRI	magnetic resonance imaging
NPV	negative predictive value
PPV	positive predictive value
PS-ECOG	Eastern Cooperative Oncology Group Performance Status
STARD	Standards for Reporting Diagnostic Accuracy
TP	true positive
